# Interaction Between Heart Rate Variability and Heart Rate in Pediatric Population

**DOI:** 10.3389/fphys.2015.00385

**Published:** 2015-12-18

**Authors:** Jakub S. Gąsior, Jerzy Sacha, Piotr J. Jeleń, Mariusz Pawłowski, Bożena Werner, Marek J. Dąbrowski

**Affiliations:** ^1^Cardiology Clinic of Physiotherapy Division of the 2nd Faculty of Medicine, Medical University of WarsawWarsaw, Poland; ^2^Faculty of Physical Education and Physiotherapy, Opole University of TechnologyOpole, Poland; ^3^Department of Biophysics and Human Physiology, Medical University of WarsawWarsaw, Poland; ^4^Department of Pediatric Cardiology and General Pediatrics, Medical University of WarsawWarsaw, Poland

**Keywords:** heart rate, heart rate variability, heart rate correction, children, time domain, frequency domain, autonomic nervous system, autonomic cardiac control

## Abstract

**Background:** Heart rate variability (HRV) is primarily heart rate (HR) dependent, and therefore, different HR may exert different impact on HRV. The objectives of the study were to evaluate the effect of HR on HRV in children and to determine whether HRV indices normalized to HR are sex- and age-related.

**Methods:** Short-term ECG recordings were performed in 346 healthy children. Standard time and frequency domain HRV parameters and HR were analyzed in four age subgroups (6–7, 8–9, 10–11, and 12–13 years old). To investigate the HR impact on HRV, standard HRV parameters were normalized to prevailing HR.

**Results:** Standard HRV measures did not differ between age subgroups, however, HR significantly decreased with subjects age and turned out to be the strongest determinant of HRV. The normalization of HRV to prevailing HR allowed to show that sex-related differences in standard HRV resulted from differences in HR between boys and girls. The normalized HRV significantly decreased with age—before the normalization this effect was masked by age-related HR alterations.

**Conclusions:** HR significantly impacts HRV in pediatric population and turns out to be the strongest determinant of all standard HRV indices. The differences in standard HRV between boys and girls result from differences in their HR. The normalized HRV is decreasing with age in healthy children and it is accompanied by the reduction of HR—as a net result, the standard HRV is constant in children at different ages. This may reflect the maturation of the autonomic nervous system.

## Introduction

During the past 30 years a large number of studies employing heart rate variability (HRV) analysis to assess cardiac autonomic function in both healthy subjects and patients with various pathological conditions have been published (Billman, 2011; Xhyheri et al., 2012). However, the vast majority of these studies did not consider an interaction between HRV and average heart rate (HR) (Billman, 2013; Sacha, 2013, 2014a,b,c; Monfredi et al., 2014). Since HRV is primarily HR dependent, different HR may exert different impact on HRV and, to some extent, may determine HRV values (Sacha and Grzeszczak, 2001; Chiu et al., 2003; Sacha and Pluta, 2005a,b, 2008; Nieminen et al., 2007; Billman, 2013; Sacha, 2013, 2014a,b,c; Monfredi et al., 2014; Billman et al., 2015). Moreover, the HRV and HR interaction results not only from physiological reasons (i.e., caused by the autonomic nervous system (ANS) activity) but also from a mathematical one—the latter arising from the non-linear, mathematical relationship between HR and R-R interval (Sacha and Grzeszczak, 2001; Sacha and Pluta, 2005a,b, 2008; Sacha, 2013, 2014a,b,c; Sacha et al., 2013a; Billman et al., 2015).

Due to this non-linearity, the same fluctuations of HR cause much higher changes of R-R intervals for the slow average HR than for the fast one, moreover, small average R-R interval cannot oscillate as much as large average R-R interval, since the R-R intervals should have become negative—this mathematically amplifies the effect of the ANS influence on HRV when the patient's average HR is changed (Sacha and Pluta, 2008; Sacha, 2013). The correction (normalization) of HRV with respect to HR should provide us with the information how relevant the HR influence on HRV really is. Several mathematical methods removing the HRV dependence (i.e., both mathematical and physiological) on HR have been proposed (Sacha and Grzeszczak, 2001; Sacha, 2013; Sacha et al., 2013a; Monfredi et al., 2014), which may help to elucidate the HR effect on HRV in physiological and pathological conditions. However, most of the studies addressing the HRV normalization for HR have been carried out in adults (Sacha and Grzeszczak, 2001; Sacha and Pluta, 2005a,b, 2008; Grant et al., 2013; Sacha, 2013, 2014a,b,c; Sacha et al., 2013a,b,c, 2014; Carter et al., 2014; Monfredi et al., 2014; Pradhapan et al., 2014; Billman et al., 2015) and, to the best of our knowledge, there is no such a study in pediatric population. This is even more important since children usually exhibit higher HR and higher variations of HR than adults and their HR gradually decreases with age (Fleming et al., 2011). Such changes in HR may imply age-related changes in HRV (Finley and Nugent, 1995; Jarrin et al., 2015). In order to properly understand the ANS development, it is critical to establish whether the HRV changes are directly dependent on age (what may be related to the maturation of ANS) or depend primary on HR that decreases with subjects age.

Therefore, the goals of the study were to explore the HR impact on HRV in children at different ages and to determine whether the HRV corrected for changes in HR is sex- and/or age-related.

## Materials and methods

### Study population

A study group consisted of 346 apparently healthy children of both sex at the age of 6–13 years. The parents or legal guardians were interviewed about children's diseases or medications—the school health records concerning children's health status were also checked. Fifteen subjects out of 346 were excluded from the analysis due to suspicion of cardiac and non-cardiac diseases or incomplete ECG data and technical errors in ECG recordings. Consequently, 331 healthy children (not taking any medications) at age of 6–13 years (median age: 10.2, interquartile range: 8.4–11.9 years; 158 boys, 173 girls) took part in the study. The children were divided into four age subgroups: 6–7, 8–9, 10–11, and 12–13 years. Each age subgroup was represented quite equally, i.e., number of children in the consecutive subgroups were as follows: 65 (boys/girls: 33/32), 88 (43/45), 101 (42/59), 77 (40/37)—respectively. All parents or legal guardians had received printed information about the study and gave their informed written consent. The study was approved by the University Bioethical Committee and followed the rules and principles of the Helsinki Declaration.

### Procedures

Parents or legal guardians were instructed that the children participating in the study should avoid intensive physical effort the day before and should eat a light breakfast as well as refrain from physical activity on the day of study examinations. The ECG recordings were carried out in a quiet school health room at least 1 h after home breakfast and before lunch. Children were asked not to eat or drink anything at least 1 h before ECG examination. All recordings were obtained during regular school days.

### ECG acquisition

Twelve-lead, 6-min ECG recordings were performed between 8 a.m. and 2 p.m. in a supine position using a portable PC with integrated software system (Custo cardio 100 12-channel PC ECG system; Custo med GmbH, Ottobrunn, Germany) working at the sampling frequency of 1000 Hz. For the purpose of stabilizing the HR before the ECG examination children were asked to lie in supine position for 5 min, and encouraged to breathe naturally as well as abstain from speaking or moving during the examination.

### HRV measurement

Prior to HRV analysis, the ECG recordings were visually inspected for potential non-sinus or aberrant beats (Peltola, 2012). The erroneous beats were manually corrected, i.e., one R-R interval before and one after each non-sinus beat were eliminated and replaced by R-R intervals computed by interpolation of degree zero based on the surrounding normal beats. HRV analysis was performed based on 5-min ECG time series using Kubios HRV 2.1 software (University of Eastern Finland, Kuopio, Finland) (Tarvainen et al., 2014). Time and frequency domain measures of HRV were computed according to Task Force of the European Society of Cardiology and the North American Society of Pacing & Electrophysiology guidelines (Task Force of the European Society of Cardiology the North American Society of Pacing Electrophysiology, 1996). The following standard time-domain measures were determined: standard deviation of all normal R-R intervals (SDNN), root mean square of successive R-R interval differences (RMSSD) and pNN50 which denotes the percent of R-R intervals differing >50 ms from the preceding one.

Before calculating spectral HRV parameters, the detrending method based on smoothness priors approach was employed (smoothing parameter, Lambda value = 1000) (Tarvainen et al., 2002). Then, R-R interval series were transformed to evenly sampled time series (4-Hz resampling rate). The detrended and interpolated R-R interval series were used to compute frequency-domain HRV measures. HRV spectra were calculated by using fast-Fourier-transform (FFT) with Welch's periodogram method (50% overlap window and 60 s window width). The following standard spectral components were distinguished: low frequency (LF, 0.04–0.15 Hz), high frequency (HF, 0.15–0.40 Hz) and total power (TP, 0.04–0.4 Hz)—all in absolute units, i.e., ms^2^ (Task Force of the European Society of Cardiology the North American Society of Pacing Electrophysiology, 1996).

### HRV correction (normalization)

To investigate the impact of HR on HRV, standard HRV parameters were normalized (i.e., corrected) with respect to an average HR. This normalization (i.e., the removing both mathematical and physiological HRV dependence on HR) relied on the division of standard HRV indices by different powers of their corresponding average R-R interval (Sacha et al., 2013a,c).

### Statistical methods

The Kolmogorov-Smirnov test was used to assess the normality of the data distribution.

Since all HRV parameters did not exhibit normal distribution, the data were presented as median and interquartile range (IQR). Spearman's rank correlation coefficient was used to assess the relationship between HRV parameters and other variables. To determine the influence of HR, age and sex on HRV, the multiple regression analysis was carried out on the condition that residuals of HRV parameters presented normal distribution. In addition, the multiple regression analysis employing logarithmically transformed HRV parameters was performed to ensure linearity of the association between HRV and HR. Mann-Whitney test was used to determine gender differences. The analysis of HRV differences between age groups was performed using Kruskal-Wallis test followed by Dunn's multiple comparisons test. The threshold probability of p < 0.05 was taken as the level of statistical significance. Statistical calculations were performed using STATISTICA 10-StatSoft. Inc. software (Tulsa, USA).

## Results

**Figure 1:**
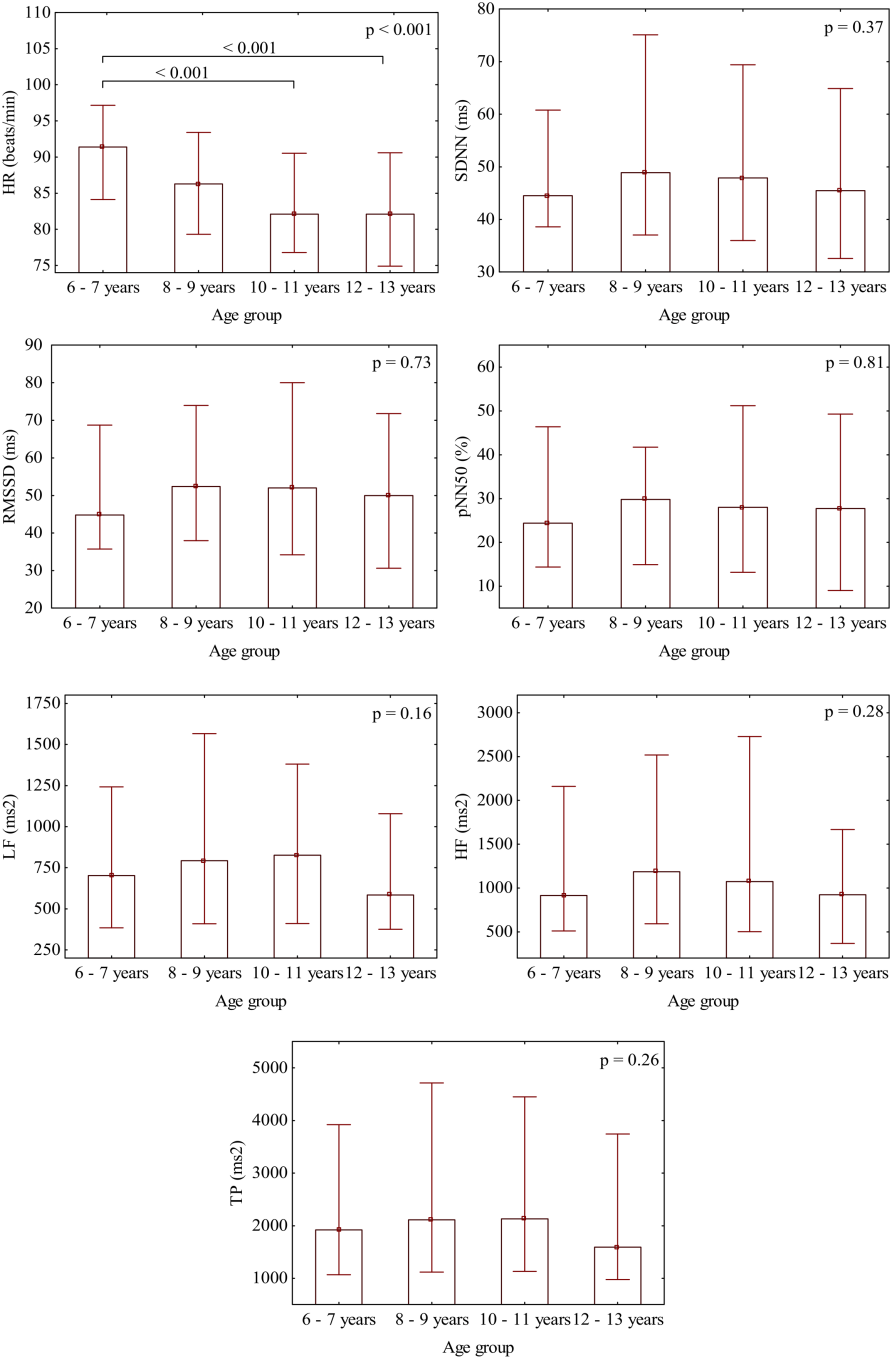
**Gradual decrease of HR with age and the distribution of the respective standard HRV parameters in each age subgroup**. Bars represent medians with whiskers indicating interquartile ranges.

**Figure 2:**
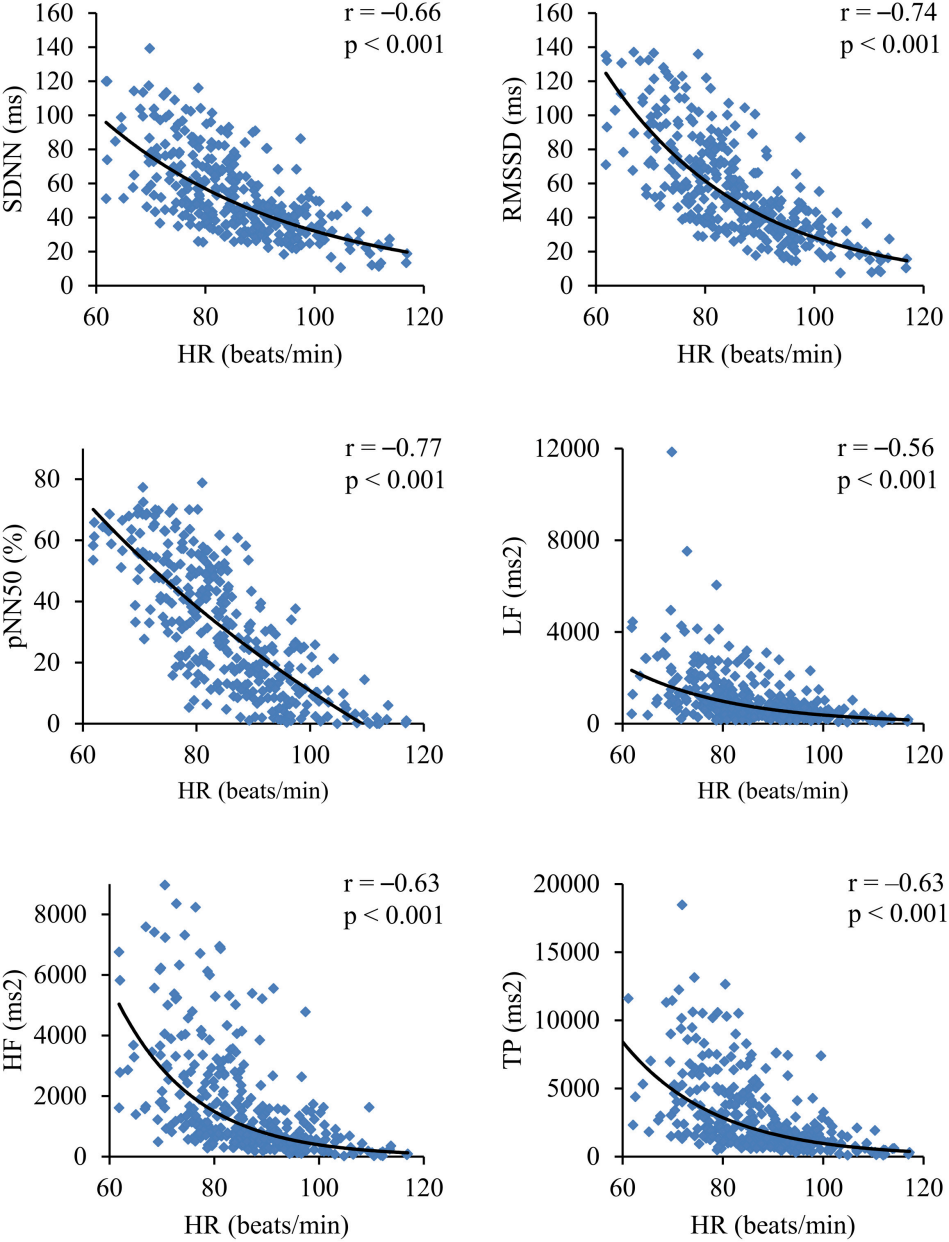
**Correlations coefficients and corresponding *p*-values between standard HRV parameters and HR**.

There was no significant difference in gender distribution between the age subgroups (*p* = 0.50). In the consecutive age subgroups the standard HRV parameters were not statistically different (Figure 1) and they did not present any relevant correlation with age, i.e., SDNN, *r* = −0.06 (*p* = 0.29); RMSSD, *r* = −0.03 (*p* = 0.59); pNN50, *r* = −0.01 (*p* = 0.88); LF, *r* = −0.06 (*p* = 0.27); HF, *r* = −0.06 (*p* = 0.29); and TP, *r* = −0.06 (*p* = 0.31). On the other hand, HR significantly differed between the age subgroups (Figure 1) and revealed a significant correlation with age (*r* = −0.24, *p* < 0.001) (**Figure 5B**). All standard HRV indices were negatively correlated with HR (Figure 2) with correlation coefficients ranging between −0.56 to −0.77 (*p* < 0.001 for all).

In the entire study group boys presented significantly higher HRV than girls in terms of any standard HRV parameter but also exhibited lower HR, i.e., the respective medians: SDNN, 52.3 ms vs. 44.8 ms (*p* < 0.01); RMSSD, 57.8 ms vs. 44.8 ms (*p* < 0.01); pNN50, 33.5% vs. 24.1% (*p* < 0.01); LF, 862.0 ms^2^ vs. 633.0 ms^2^ (*p* < 0.01); HF, 1328.0 ms^2^ vs. 925.0 ms^2^ (*p* < 0.05); TP, 2281.0 ms^2^ vs. 1784.0 ms^2^ (*p* < 0.01) and HR: 82.5 bpm vs. 87.0 bpm (*p* < 0.001).

In the multiple regression analysis HR turned out to be the strongest determinant of all analyzed standard HRV with β-value ranging between −0.48 and −0.79, however, no standard HRV parameter was independently associated with sex (Table 1)—the models accounted for 22–57% of the HRV variance (see determination coefficients).

**Table 1:** **Multiple regression analysis—determinants of standard HRV parameters**.

**Standard HRV parameter**	**Determinant**	**Parameters of multiple regression analysis**				
		**β-value**	***p*-value**	**Multiple *R*^2^**	***F*-test**	***p*-value**
SDNN (ms)	HR	−0.64	< 0.001	0.42	119.4	< 0.001
	SEX	0.04	0.41			
RMSSD (ms)	HR	−0.71	< 0.001	0.52	175.8	< 0.001
	SEX	0.03	0.46			
pNN50 (%)	HR	−0.75	< 0.001	0.57	217.2	< 0.001
	SEX	0.02	0.59			
LF (ms^2^)	HR	−0.45	< 0.001	0.22	47.0	< 0.001
	SEX	0.09	0.06			
HF (ms^2^)	HR	−0.53	< 0.001	0.27	62.1	< 0.001
	SEX	−0.02	0.75			
TP (ms^2^)	HR	−0.54	< 0.001	0.30	71.0	< 0.001
	SEX	0.03	0.52			

The analyses were performed on the raw data (residuals of all HRV parameters presented normal distribution)—additional multiple regression analyses based on logarithmically transformed HRV parameters yielded exactly the same results.

**Figure 3:**
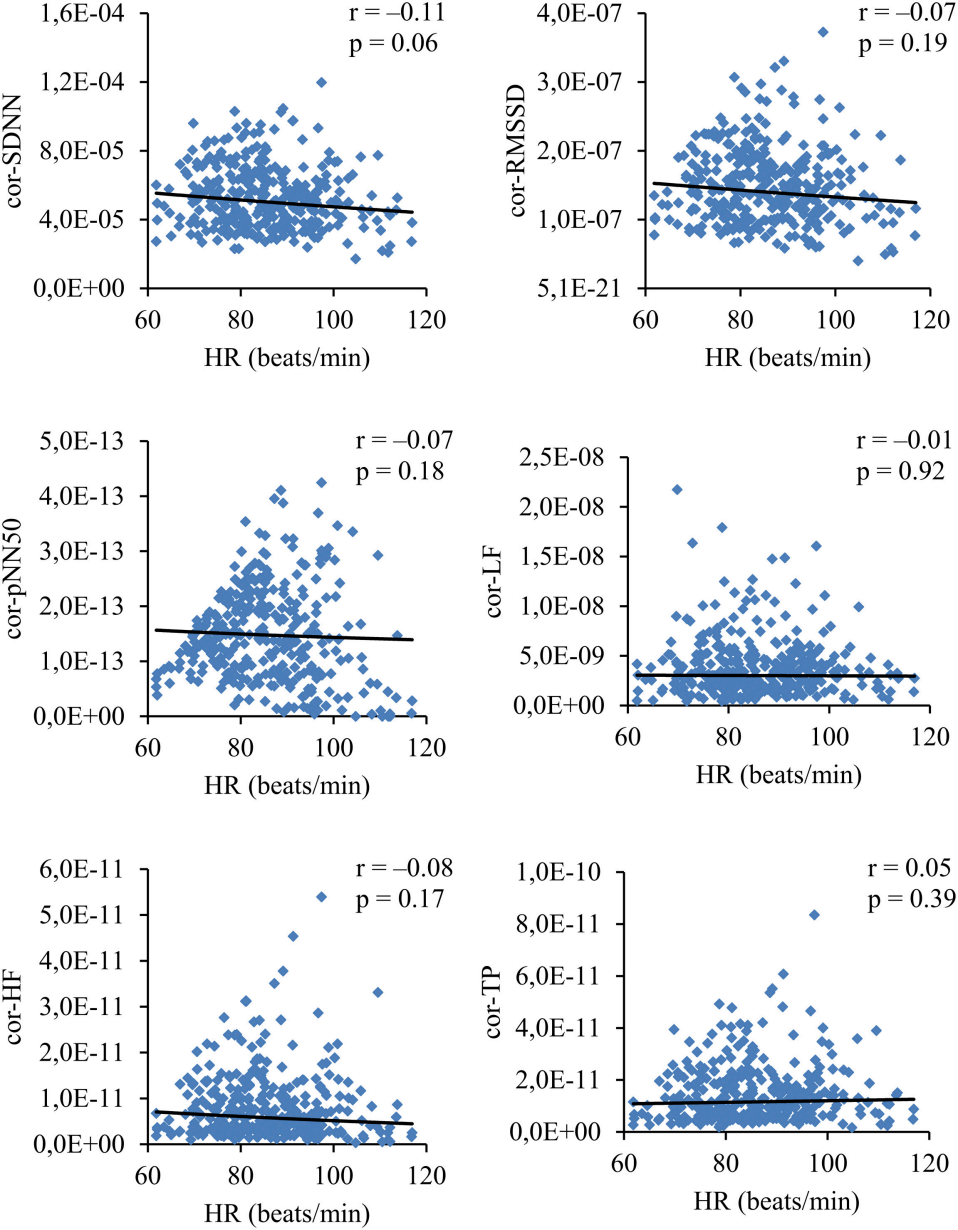
**Correlations coefficients and corresponding *p*-values between HR and normalized (i.e., corrected for HR) HRV indices**.

**Figure 4:**
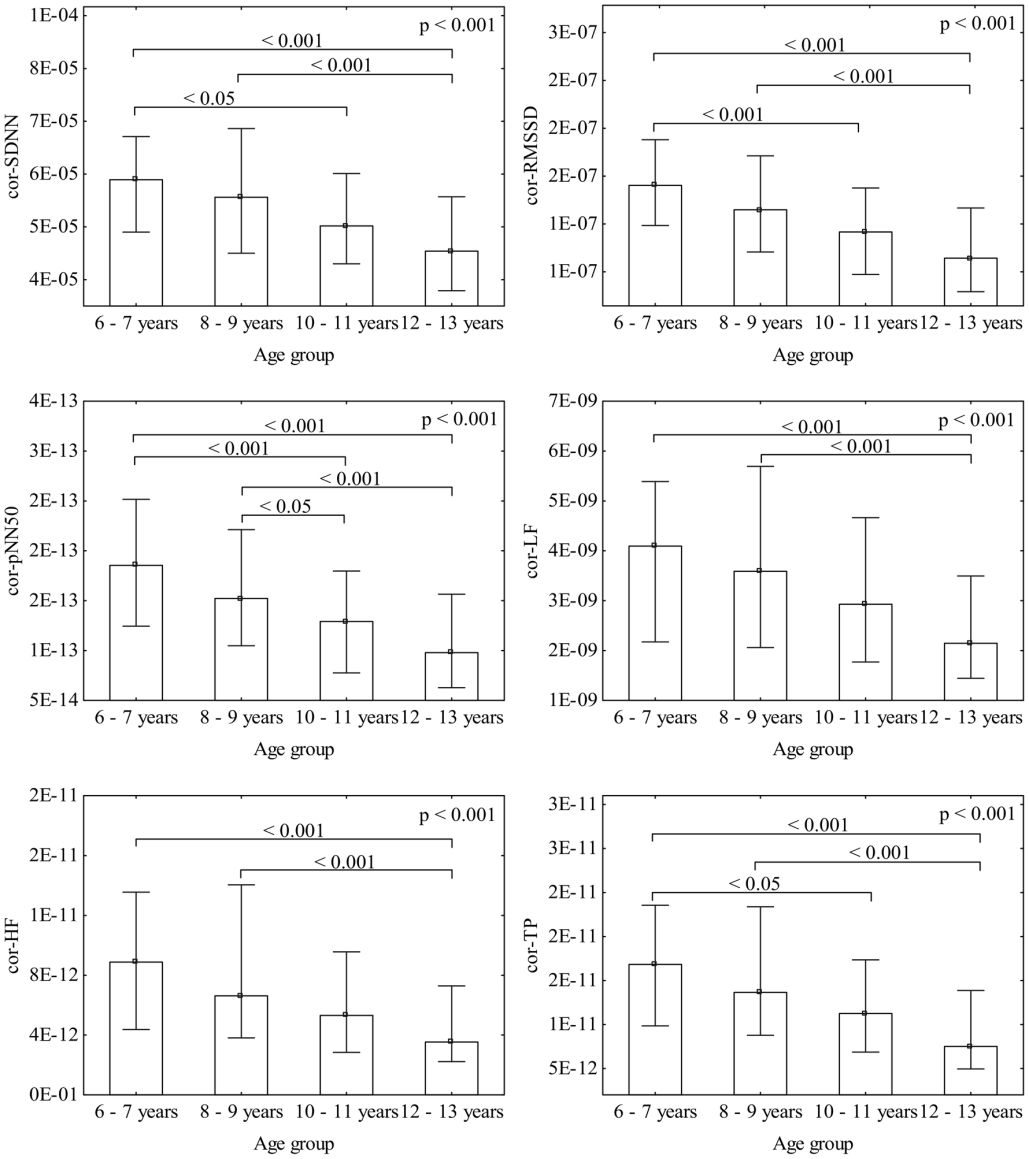
**Gradual decrease of the respective normalized HRV parameters with age**. Bars represent medians with whiskers indicating interquartile ranges.

To exclude the overall HR impact on HRV, the respective parameters were normalized for HR. HRV lost their dependence on HR after dividing SDNN, RMSSD, pNN50, LF, HF and TP by (respectively): avRR^∧^2.1, avRR^∧^3.0, avRR^∧^5.0, avRR^∧^4.0, avRR^∧^5.0, and avRR^∧^5.0 (Figure 3). The normalized HRV (i.e., independent on HR) decreased in the consecutive age subgroups (Figure 4). Moreover, all normalized HRV indices revealed significant negative correlations with age: cor-SDNN, *r* = −0.28; cor-RMSSD, *r* = −0.34; cor-pNN50, *r* = −0.31; cor-LF, *r* = −0.24; cor-HF, *r* = −0.28; cor-TP, *r* = −0.29 (*p* < 0.001 for all). In other words, the normalized HRV significantly attenuated with children's age. The results did not reveal any significant differences between boys and girls in their normalized HRV parameters (*p* > 0.26 for all). The additional multiple regression analysis revealed that age was the only independent determinant of normalized HRV (Table 2).

**Table 2:** **Multiple regression analysis—determinants of corrected HRV parameters**.

**Corrected HRV parameter**	**Determinant**	**Parameters of multiple regression analysis**				
		**β-value**	***p*-value**	**Multiple *R*^2^**	***F*-test**	***p*-value**
cor-SDNN	AGE	−0.27	< 0.001	0.08	13.8	< 0.001
	SEX	0.06	0.29			
cor-RMSSD	AGE	−0.32	< 0.001	0.10	18.6	< 0.001
	SEX	0.05	0.36			
cor-pNN50	AGE	−0.31	< 0.001	0.10	17.3	< 0.001
	SEX	0.03	0.56			
cor-LF	AGE	−0.22	< 0.001	0.06	10.0	< 0.001
	SEX	0.10	0.08			
cor-HF	AGE	−0.21	< 0.001	0.04	7.5	< 0.001
	SEX	−0.02	0.70			
cor-TP	AGE	−0.26	< 0.001	0.07	11.4	< 0.001
	SEX	0.01	0.90			

The analyses were performed on the raw data (residuals of all HRV parameters presented normal distribution)—additional multiple regression analyses based on logarithmically transformed HRV parameters yielded exactly the same results.

## Discussion

**Figure 5:**
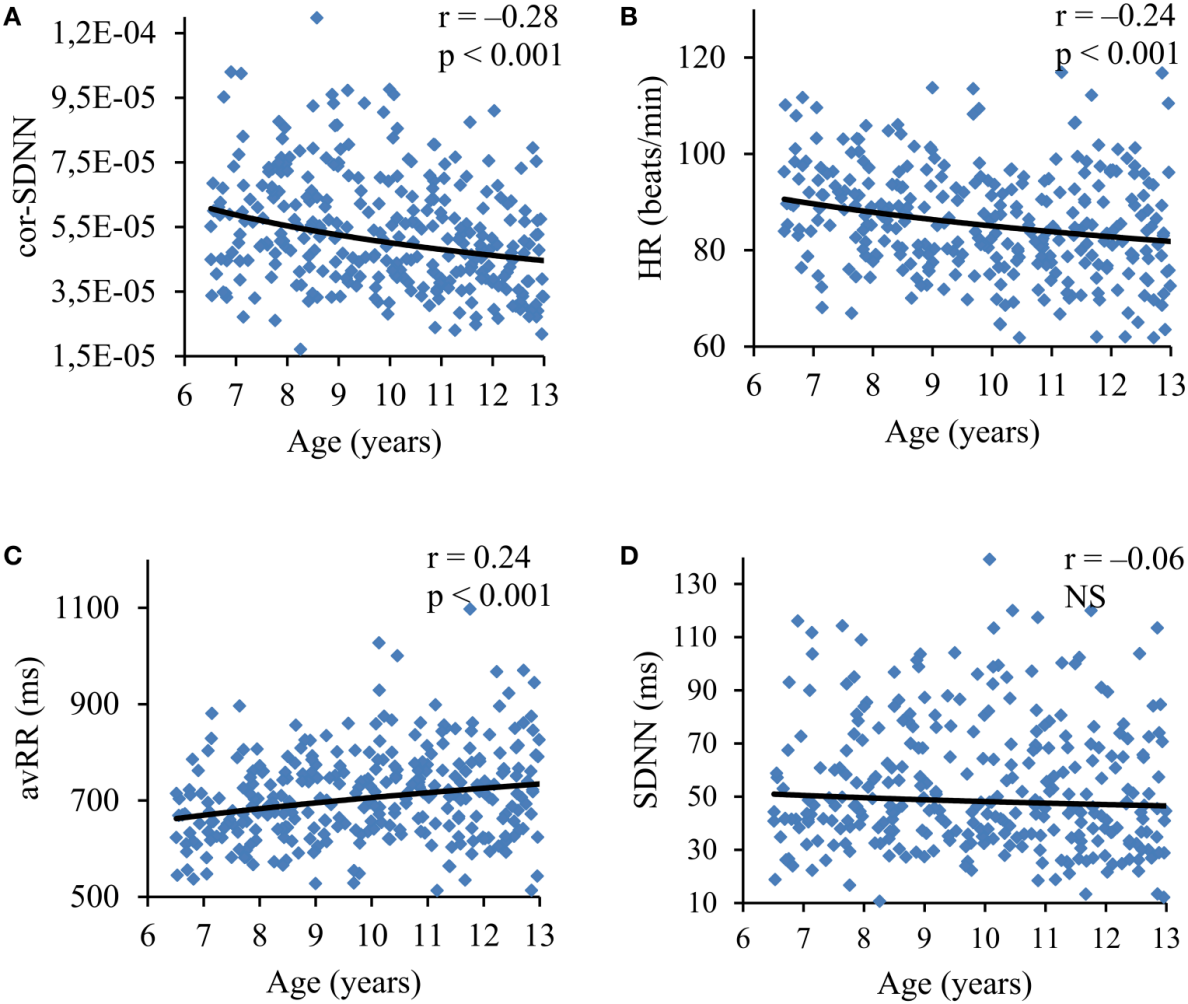
**The relation of normalized SDNN (cor-SDNN) (A), average heart rate (HR) (B), average R-R interval (avRR) (C), and standard SDNN (SDNN) (D) with children's age**. One can see that cor-SDNN and HR decrease (avRR increases) with age—as a net result, standard SDNN is almost constant.

The main finding of our study is that the normalized HRV (i.e., corrected for prevailing HR) decreases with age in healthy children between 6 and 13 years and this is accompanied by a reduction of average HR (i.e., an increase in average R-R interval)—as a net result, the standard HRV (i.e., before correction for HR) is constant in children at different age in our group (Figure 5 summarizes these findings). As far as we know, this is the first observation demonstrating that higher HR in younger children is accompanied by higher normalized variability (i.e., higher corrected HRV), whereas lower HR in older children is accompanied by the reduction of normalized variability. Moreover, differences in standard HRV between boys and girls actually result from differences in their HR. In fact, all standard HRV indices are significantly associated with HR (Figure 2, Table 1). Among common variables, such as age, sex and HR, the latter turns out to be the strongest determinant for all standard HRV parameters.

A growing number of studies on HRV demonstrate that HR has to be considered in the interpretation of HRV analysis, especially when study subjects differ in terms of their average HR (Sacha and Grzeszczak, 2001; Sacha and Pluta, 2005a,b, 2008; Billman, 2013; Grant et al., 2013; Sacha, 2013, 2014a,b,c; Sacha et al., 2013a,b,c, 2014; Carter et al., 2014; Monfredi et al., 2014; Pradhapan et al., 2014; Billman et al., 2015). As demonstrated previously, HR influences standard HRV due to both physiological and mathematical reasons (Sacha and Grzeszczak, 2001; Sacha and Pluta, 2005a,b, 2008; Sacha, 2013, 2014a,b,c; Sacha et al., 2013a,b,c, 2014; Monfredi et al., 2014; Billman et al., 2015). Consequently, the subjects with slow HR usually present higher standard HRV, but those with fast HR commonly exhibit lower standard HRV (Sacha and Grzeszczak, 2001; Sacha and Pluta, 2005a,b, 2008; Billman, 2013; Sacha, 2013, 2014a,b,c; Sacha et al., 2013a,b,c, 2014; Monfredi et al., 2014; Billman et al., 2015)—such a negative correlation between standard HRV and HR could also be observed in our study group (Figure 2). It is therefore reasonable to correct HRV for prevailing HR before drawing conclusions about HRV (Sacha, 2013; Sacha et al., 2013a; Monfredi et al., 2014; Billman et al., 2015). Furthermore, there is evidence that such correction may improve the HRV prognostic value for some outcomes in certain patient populations, e.g., those with ischemic heart disease and after myocardial infarction (Sacha et al., 2013b, 2014; Pradhapan et al., 2014). In fact, standard HRV provides information on two quantities, i.e., on HR and its variability. It is hard to determine which of these two plays a principal role in the clinical value of HRV—therefore, it is critical to separate information coming from HR and its variability (Sacha et al., 2013a,b,c, 2014; Sacha, 2014a,b,c). The HR normalization of HRV (which removes both mathematical and physiological HRV and HR dependence) may enable to explore the HR influence on the prognostic power of HRV in both adults and children.

Another point supporting the removal of the HR impact on HRV is the sex-related difference in HR (Nanchen et al., 2013; Sacha et al., 2014), which can also be observed in children, i.e., girls usually exhibit higher HR and lower standard HRV than boys (Silvetti et al., 2001; Faulkner et al., 2003; Moodithaya and Avadhany, 2012; Michels et al., 2013; Jarrin et al., 2015). After the normalization for HR, HRV did not differ by gender in our study group. This suggests that previously reported sex-related differences in standard HRV in children (Silvetti et al., 2001; Faulkner et al., 2003; Moodithaya and Avadhany, 2012; Michels et al., 2013; Jarrin et al., 2015) could result from differences in HR. In the multiple regression analysis HR turned out to be the only significant determinant of standard HRV, whereas sex had no independent influence on the variability (Table 1).

HR gradually declines with growing in either sex (Finley and Nugent, 1995; Goto et al., 1997; Fleming et al., 2011; O'Leary et al., 2015). Importantly, such developmental HR alterations may lead to an augmentation of standard HRV, which in turn can be hastily interpreted as HRV changes associated with age. In the studies where standard HRV increased with age, a parallel decrease in HR (i.e., an increase in average R-R interval) was also observed (Goto et al., 1997; Massin and von Bernuth, 1997; Pikkujämsä et al., 1999; Silvetti et al., 2001; Cysarz et al., 2011, 2013). Dependence of HRV on HR in children was already reported two decades ago when significant correlation between mean R-R interval and standard HRV indices in Holter recordings was noticed (Goto et al., 1997; Massin and von Bernuth, 1997). However, to the best of our knowledge, no previous study examined age-related changes in HRV independently of HR. This is a critical issue, since HR variations may mask a real association between age and HRV. Children in our group presented HR decreasing with age (Figures 1, 5B), nonetheless, their standard HRV was constant (Figures 1, 5D). After the exclusion of HR impact, we were able to detect age-related changes in HRV, i.e., the normalized HRV indices revealed negative correlations with age and significantly decreased in the consecutive age subgroups (Figures 4, 5A). Additionally, in the multiple regression analysis the normalized HRV proved to be independently associated with age (Table 2). We do not know why the normalized variability decreased with age, however, this was accompanied by the decrease in average HR, and consequently standard HRV did not change at various ages. Indeed, standard HRV remains constant, if the normalized variability is higher when HR is fast in younger children, and if it decreases when HR slows down in older children—such a phenomenon was observed in our study (Figure 5). This observation may shed new light on the maturation of autonomic nervous system, nevertheless, it is hard to draw any solid conclusions about a possible physiological mechanism responsible for this phenomenon and further studies are necessary to prove these findings in broader age populations.

## Limitations

Some limitations of our work need to be recognized. First, the effect of age on HRV was not analyzed among the same subjects followed for years, but in different children at different ages. Thus, the conclusions concerning the HRV changes during children growth are based on indirect observation. Second, we investigated relatively narrow age range, i.e., from 6 to 13 year-olds, hence, we do not know how HRV and HR behave beyond this age range—the development of the autonomic nervous system certainly begins earlier and lasts longer.

## Conclusion

HR significantly impacts HRV in pediatric population and turns out to be the strongest, independent determinant of all standard HRV indices. Moreover, the differences in standard HRV between boys and girls actually result from differences in their HR. As a result of the HRV normalization with respect to HR, one may uncover the association between the normalized HRV and children's age. Indeed, the normalized HRV is decreasing with age in healthy children and it is accompanied by the reduction of average HR—as a net result, the standard HRV is constant in children at different ages in our study group.

## Author contributions

Conceived and designed the experiment: JG, PJ, MP, MD. The acquisition, analysis, or interpretation of data for the work: JG, JS, PJ, MP, BW, MD. Drafting the work or revising it critically for important intellectual content: JG, JS, PJ, MP, BW, MD. Final approval of the version to be published: JG, JS, PJ, MP, BW, MD. Agreement to be accountable for all aspects of the work in ensuring that questions related to the accuracy or integrity of any part of the work are appropriately investigated and resolved: JG, JS, PJ, MP, BW, MD.

## Funding

The authors and clinical organizations with which the authors are affiliated or associated did not receive any grants or outside funding in support of the research for or preparation of the manuscript, did not receive payments or other benefits from a commercial entity, do not have any professional relationships with companies or manufacturers who will benefit from the results of the present study. The aforementioned disclosure also applies to the authors' immediate families.

### Conflict of interest statement

The authors declare that the research was conducted in the absence of any commercial or financial relationships that could be construed as a potential conflict of interest.
